# A class of fourth-order parabolic equation with logarithmic nonlinearity

**DOI:** 10.1186/s13660-018-1920-7

**Published:** 2018-11-27

**Authors:** Pingping Li, Changchun Liu

**Affiliations:** Department of Mathematics, Jilin University, Changchun, China

**Keywords:** 35K35, 35A01, 35B44, 35K55, Global existence, Blow-up, Logarithmic source term

## Abstract

In this paper, we study a class of fourth-order parabolic equation with the logarithmic nonlinearity. By using the potential well method, we obtain the existence of the unique global weak solution. In addition, we also obtain results of decay and blow-up in the finite time for the weak solution.

## Introduction

In this paper, we study the following problem:

1.1 $$\begin{aligned} \textstyle\begin{cases} u_{t}+\Delta^{2}u= \vert u \vert ^{q-2}u\log \vert u \vert ,\quad x\in\varOmega, t>0, \\ u(x,t)=\Delta u(x,t)=0,\quad x\in\partial\varOmega, t>0, \\ u(x,0)=u_{0}(x),\quad x\in\varOmega, \end{cases}\displaystyle \end{aligned}$$

where *Ω* is a bound domain in $\mathbb {R}^{n}$ with smooth boundary, $2< q<2+\frac{4}{n}$, $u_{0}(x)\in H_{0}^{2}(\varOmega)\setminus\{0\}$.

Many papers have been devoted to the fourth-order parabolic equation. Qu and Zhou [1] studied the following fourth-order equation:

1.2 $$ u_{t}+D^{4}u= \vert u \vert ^{p-1}u- \frac{1}{ \vert \varOmega \vert } \int_{\varOmega } \vert u \vert ^{p-1}u\,dx. $$

Using the method of potential wells, they established a threshold result for the global existence and blow-up for the sign-changing weak solutions. Zhou [2] proved new blow-up conditions and the maximum of the blow-up time for Eq. (1.2). Li, Gao and Han [3] considered

$$\begin{aligned} \textstyle\begin{cases} u_{t}+D^{4}u- ( \vert u_{x} \vert ^{p-2}u_{x} )_{x}= \vert u \vert ^{p-1}u- \frac{1}{ \vert \varOmega \vert } \int _{\varOmega} \vert u \vert ^{p-1}u\,dx,\quad x\in\varOmega, \\ Du=D^{3}u=0,\qquad (x,t)\in\partial\varOmega\times(0,T), \\ u(x,0)=u_{0}(x),\quad x\in\varOmega. \end{cases}\displaystyle \end{aligned}$$

They obtained the existence, uniqueness and blow-up of solutions. Liu and Liu [4] considered the following equation:

$$\begin{aligned} &u_{t}-D^{6}u+D \biggl(a(Du)D^{3}u+ \frac{a'(Du)}{2} \bigl(D^{2}u \bigr)^{2} \biggr) \\ &\quad = \vert u \vert ^{p-1}u-\frac{1}{ \vert \varOmega \vert } \int_{\varOmega} \vert u \vert ^{p-1}u\,dx, \quad (x,t) \in\varOmega \times(0,T). \end{aligned}$$

They combine the potential well method, the classical Galerkin method and the energy method to give a threshold result for the global existence and non-existence of sign-changing weak solutions to the problem. The relevant equations have also been studied in [5, 6].

In this paper, we study a fourth-order parabolic equation with the logarithmic nonlinearity. The second-order parabolic equation with the logarithmic nonlinearity is diffusely studied. Chen, Luo and Liu [7] studied the heat equation with the logarithmic nonlinearity. Ji, Yin and Cao [8] established the existence of positive periodic solutions and discussed the instability of such solutions for the semilinear pseudo-parabolic equation with the logarithmic source. Nahn and Truong [9] studied the following nonlinear equation:

1.3 $$ u_{t}-\Delta u_{t}-\operatorname{div} \bigl( \vert \nabla u \vert ^{p-2}\nabla u \bigr)= \vert u \vert ^{p-2}u\log\bigl( \vert u \vert \bigr). $$

They obtained results as regards the existence or non-existence of global weak solutions. He, Gao and Wang [10] considered the following equation:

1.4 $$ u_{t}-\Delta u_{t}-\operatorname{div} \bigl( \vert \nabla u \vert ^{p-2}\nabla u \bigr)= \vert u \vert ^{q-2}u\log\bigl( \vert u \vert \bigr), $$

where $2< p< q< p(1+\frac{2}{n})$, they proved the decay and the finite time blow-up for weak solutions.

In this paper, we prove the existence of the unique global weak solution of the problem (1.1) based on the potential well method. In addition, we also obtain some properties of the solutions. This paper is organized as follows: in Sect. 2, we introduce some lemmas. In Sect. 3, we mainly introduce the existence of the unique local weak solution of the problem (1.1). In Sect. 4, under some conditions, we obtain the existence of the unique global weak solution of the problem (1.1). Meanwhile, we find that the solution is decaying. In the last section, we prove the blow-up theorem.

## Some lemmas

We first consider the energy functional *J* and Nehari functional *I* defined on $H_{0}^{2}(\varOmega)\setminus\{0\}$ as follows:

2.1 $$\begin{aligned} &J(u)=\frac{1}{2} \int_{\varOmega} \vert \Delta u \vert ^{2}\,dx- \frac{1}{q} \int_{\varOmega} \vert u \vert ^{q}\log \vert u \vert \,dx+ \frac{1}{q^{2}} \int_{\varOmega} \vert u \vert ^{q}\,dx, \end{aligned}$$

2.2 $$\begin{aligned} &I(u)= \int_{\varOmega} \vert \Delta u \vert ^{2}\,dx- \int_{\varOmega} \vert u \vert ^{q}\log \vert u \vert \,dx. \end{aligned}$$

We can see that *J* and *I* are continuous from the Gagliardo–Nirenberg multiplicative embedding inequality (see [11]).

By (2.1) and (2.2), we have

2.3 $$\begin{aligned} &J(u)=\frac{1}{q}I(u)+ \biggl(\frac{1}{2}- \frac{1}{q} \biggr) \int_{\varOmega} \vert \Delta u \vert ^{2}\,dx+ \frac{1}{q^{2}} \int_{\varOmega} \vert u \vert ^{q}\,dx. \end{aligned}$$

Let $\mathscr{N}=\{u\in H_{0}^{2}(\varOmega)\setminus\{0\}:I(u)=0\}$. Lemma 2.1 indicates $\mathscr{N}$ is not empty. Thus, we can define

2.4 $$\begin{aligned} d=\inf_{u\in\mathscr{N}}J(u). \end{aligned}$$

It is obvious that $d>0$ by (2.3), (2.4), $2< q<2+\frac {4}{n}$ and the definition of $\mathscr{N}$. For a fixed $u\in H_{0}^{2}(\varOmega)\setminus\{0\}$, we consider the function $j:\lambda\rightarrow J(\lambda u)$ for $\lambda>0$.

### Lemma 2.1

*Let* $u\in H_{0}^{2}(\varOmega)\setminus\{0\}$. *Then the following results hold*:

1. $\lim_{\lambda\to0^{+}}j(\lambda)=0, \lim_{\lambda\to+\infty }j(\lambda)=0$;
2. *there exists a unique* $\overline{\lambda}>0$ *such that* $j'(\overline {\lambda})=0$;
3. $j(\lambda)$ *is increasing on* $(0,\overline{\lambda})$, *decreasing on* $(\overline{\lambda},+\infty)$ *and attains the maximum at* *λ̅*;
4. $I(\lambda u)>0$ *for* $0<\lambda<\overline{\lambda}$, $I(\lambda u)<0$ *for* $\overline{\lambda}<\lambda<+\infty$ *and* $I(\overline{\lambda}u)=0$.

### Proof

For $u\in H_{0}^{2}(\varOmega)\setminus\{0\}$, by the definition of *j*, we have

$$\begin{aligned} j(\lambda)=J(\lambda u)={}&\frac{1}{2} \int_{\varOmega}\bigl\vert \Delta(\lambda u) \bigr\vert ^{2}\,dx- \frac{1}{q} \int_{\varOmega} \vert \lambda u \vert ^{q}\log \vert \lambda u \vert \,dx+ \frac{1}{q^{2}} \int_{\varOmega} \vert \lambda u \vert ^{q}\,dx \\ ={}&\frac{\lambda^{2}}{2} \int_{\varOmega} \vert \Delta u \vert ^{2}\,dx- \frac{\lambda ^{q}}{q} \int_{\varOmega} \vert u \vert ^{q}\log \vert u \vert \,dx- \frac{\lambda^{q}}{q}\log \lambda \int_{\varOmega} \vert u \vert ^{q}\,dx \\ &{}+\frac{\lambda^{q}}{q^{2}} \int_{\varOmega} \vert u \vert ^{q}\,dx. \end{aligned}$$

It is obvious that (1) holds due to $2< q<2+\frac{4}{n}$ and $\int _{\varOmega}|u|^{q}\,dx\neq0$. We have

$$\begin{aligned} j'({\lambda})={}&\lambda \int_{\varOmega} \vert \Delta u \vert ^{2}\,dx- \lambda^{q-1} \int _{\varOmega} \vert u \vert ^{q}\log \vert u \vert \,dx -\lambda^{q-1} \log\lambda \int_{\varOmega} \vert u \vert ^{q}\,dx \\ &{}-\frac{\lambda^{q-1}}{q} \int_{\varOmega} \vert u \vert ^{q}\,dx + \frac{\lambda^{q-1}}{q} \int_{\varOmega} \vert u \vert ^{q}\,dx \\ ={}&\lambda \int_{\varOmega} \vert \Delta u \vert ^{2}\,dx- \lambda^{q-1} \int_{\varOmega} \vert u \vert ^{q}\log \vert u \vert \,dx -\lambda^{q-1} \log\lambda \int_{\varOmega} \vert u \vert ^{q}\,dx. \end{aligned}$$

We construct a function $\varphi(\lambda)=\lambda^{-1}j'(\lambda)$, thus we obtain

$$\begin{aligned} \varphi(\lambda)={}&\lambda^{-1}j'(\lambda) \\ ={}&\lambda^{-1} \biggl(\lambda \int_{\varOmega} \vert \Delta u \vert ^{2}\,dx-\lambda ^{q-1} \int_{\varOmega} \vert u \vert ^{q}\log \vert u \vert \,dx -\lambda^{q-1} \log\lambda \int_{\varOmega} \vert u \vert ^{q}\,dx \biggr) \\ ={}& \int_{\varOmega} \vert \Delta u \vert ^{2}\,dx- \lambda^{q-2} \int_{\varOmega} \vert u \vert ^{q}\log \vert u \vert \,dx -\lambda^{q-2} \log\lambda \int_{\varOmega} \vert u \vert ^{q}\,dx. \end{aligned}$$

Then

$$\begin{aligned} &\varphi'(\lambda)=-(q-2)\lambda^{q-3} \int_{\varOmega} \vert u \vert ^{q}\log \vert u \vert \,dx \\ &\qquad{}-(q-2)\lambda^{q-3}\log\lambda \int_{\varOmega} \vert u \vert ^{q}\,dx - \lambda^{q-3} \int_{\varOmega} \vert u \vert ^{q}\,dx \\ &\quad =-\lambda^{q-3} \biggl((q-2) \int_{\varOmega} \vert u \vert ^{q}\log \vert u \vert \,dx +(q-2)\log\lambda \int_{\varOmega} \vert u \vert ^{q}\,dx+ \int_{\varOmega} \vert u \vert ^{q}\,dx \biggr), \end{aligned}$$

which implies that there exists a $\lambda_{1}>0 $ such that $\varphi (\lambda)$ is increasing on $(0,\lambda_{1})$, decreasing on $(\lambda _{1},+\infty)$. Using the Poincaré inequality and $u\in H_{0}^{2}(\varOmega)\setminus\{ 0\}$, we have $0<\int_{\varOmega}|u|^{2}\,dx\leq C_{1}\int_{\varOmega}|Du|^{2}\,dx\leq C_{1}C_{2}\int _{\varOmega}|\Delta u|^{2}\,dx$, where $C_{1},C_{2}$ is the Poincaré constants. Since $\lim_{\lambda\to0^{+}}\varphi(\lambda)=\int_{\varOmega}|\Delta u|^{2}\,dx>0$, $\lim_{\lambda\to+\infty}\varphi(\lambda)=-\infty$, there exists a unique $\overline{\lambda}>0$ such that $\varphi(\overline {\lambda})=0$, i.e. $j'(\overline{\lambda})=0$. So (2) holds. Thus $j'(\lambda)=\lambda\varphi(\lambda)>0$ for $0<\lambda<\overline{\lambda }$ and $j'(\lambda)<0$ for $\overline{\lambda}<\lambda<+\infty$, which indicates $j(\lambda)$ is increasing on $(0,\overline{\lambda})$, decreasing on $(\overline {\lambda},+\infty)$ and attains the maximum at *λ̅*. So (3) holds. From (2.2), we have

$$\begin{aligned} I(\lambda u)={}& \int_{\varOmega}\bigl\vert \Delta(\lambda u) \bigr\vert ^{2}\,dx- \int_{\varOmega} \vert \lambda u \vert ^{q}\log \vert \lambda u \vert \,dx \\ ={}&\lambda^{2} \int_{\varOmega} \vert \Delta u \vert ^{2}\,dx- \lambda^{q} \int_{\varOmega} \vert u \vert ^{q}\log \vert u \vert \,dx -\lambda^{q} \log\lambda \int_{\varOmega} \vert u \vert ^{q}\,dx \\ ={}&\lambda \biggl(\lambda \int_{\varOmega} \vert \Delta u \vert ^{2}\,dx- \lambda^{q-1} \int _{\varOmega} \vert u \vert ^{q}\log \vert u \vert \,dx -\lambda^{q-1} \log\lambda \int_{\varOmega} \vert u \vert ^{q}\,dx \biggr) \\ ={}&\lambda j'(\lambda). \end{aligned}$$

Thus, $I(\lambda u)>0$ for $0<\lambda<\overline{\lambda}$, $I(\lambda u)<0$ for $\overline{\lambda}<\lambda<+\infty$ and $I(\overline{\lambda}u)=0$. So (4) holds. □

### Lemma 2.2

*There exists a* $u>0$ *with* $u\in\mathscr{N}$ *such that* $J(u)=d$.

### Proof

By (2.4), we suppose $\{u_{k}\}_{k=1}^{\infty}\subset\mathscr{N}$ is a minimizing sequence of *J*. Since $\{|u_{k}|\}_{k=1}^{\infty}\subset \mathscr{N}$ is also a minimizing sequence of *J*, we consider the case where $u_{k}>0$ a.e. in *Ω*, $k\in\mathbb {N}$ without loss of generality. Thus,

2.5 $$\begin{aligned} \lim_{k \to\infty}J(u_{k})=d, \end{aligned}$$

which implies that $\{J(u_{k})\}_{k=1}^{\infty}$ is bounded, i.e. there exists a constant $C_{3}>0$ such that $|J(u_{k})|\leq C_{3}$. Using (2.3), $I(u_{k})=0$ and $|J(u_{k})|\leq C_{3}$, we have

2.6 $$\begin{aligned} \biggl(\frac{1}{2}-\frac{1}{q} \biggr) \int_{\varOmega} \vert \Delta u_{k} \vert ^{2}\,dx+ \frac{1}{q^{2}} \int_{\varOmega} \vert u_{k} \vert ^{q}\,dx \leq C_{3}. \end{aligned}$$

Combining $2< q<2+\frac{4}{n}$ with (2.6), we have

2.7 $$\begin{aligned} \int_{\varOmega} \vert \Delta u_{k} \vert ^{2}\,dx\leq \biggl(\frac{1}{2}-\frac{1}{q} \biggr)^{-1}C_{3}. \end{aligned}$$

Using (2.7) and the Poincaré inequality, we have

$$\begin{aligned} \int_{\varOmega} \vert u_{k} \vert ^{2}\,dx \leq C_{4} \int_{\varOmega} \vert Du_{k} \vert ^{2}\,dx \leq C_{4}C_{5} \int _{\varOmega} \vert \Delta u_{k} \vert ^{2}\,dx\leq \biggl(\frac{1}{2}-\frac{1}{q} \biggr)^{-1}C_{3}C_{4}C_{5}, \end{aligned}$$

where $C_{4}, C_{5}$ are the Poincaré constants. The above inequality implies that $\{u_{k}\}_{k=1}^{\infty}$ is bounded in $H_{0}^{2}(\varOmega)$. Let $\mu_{1}>0$ be sufficiently small such that $q+\mu_{1}<\frac{2n}{n-2}$. Since $H_{0}^{2}(\varOmega)\hookrightarrow L^{q+\mu_{1}}(\varOmega)$ is a compact embedding, there exist a function *u* and a subsequence of $\{ u_{k}\}_{k=1}^{\infty}$, still denoted by $\{u_{k}\}_{k=1}^{\infty}$, such that

$$\begin{aligned} &u_{k}\rightarrow u, \quad\text{weakly in } H_{0}^{2}( \varOmega), \\ &u_{k}\rightarrow u, \quad\text{strongly in } L^{q+\mu_{1}}( \varOmega), \\ &u_{k}\rightarrow u(x),\quad \text{a.e. in } \varOmega. \end{aligned}$$

Then we have $u\geq0$ a.e. in *Ω*. First, we prove $u\neq0$. Using the dominated convergence theorem, we obtain

2.8 $$\begin{aligned} & \int_{\varOmega} \vert u \vert ^{q}\log \vert u \vert \,dx=\lim_{k \to\infty} \int_{\varOmega} \vert u_{k} \vert ^{q} \log \vert u_{k} \vert \,dx, \end{aligned}$$

2.9 $$\begin{aligned} & \int_{\varOmega} \vert u \vert ^{q}\,dx=\lim _{k \to\infty} \int_{\varOmega} \vert u_{k} \vert ^{q} \,dx. \end{aligned}$$

It follows from the weak lower semicontinuity of the $L^{2}$ norm that

2.10 $$\begin{aligned} \int_{\varOmega} \vert \Delta u \vert ^{2}\,dx\leq \liminf_{k \to\infty} \int_{\varOmega} \vert \Delta u_{k} \vert ^{2}\,dx. \end{aligned}$$

Using (2.1), (2.5), (2.8), (2.9) and (2.10), we have

2.11 $$\begin{aligned} J(u)={}&\frac{1}{2} \int_{\varOmega} \vert \Delta u \vert ^{2}\,dx- \frac{1}{q} \int_{\varOmega} \vert u \vert ^{q}\log \vert u \vert \,dx+ \frac{1}{q^{2}} \int_{\varOmega} \vert u \vert ^{q}\,dx \\ \leq{}&\liminf_{k \to\infty}\frac{1}{2} \int_{\varOmega} \vert \Delta u_{k} \vert ^{2}\,dx -\lim_{k \to\infty}\frac{1}{q} \int_{\varOmega} \vert u_{k} \vert ^{q} \log \vert u_{k} \vert \,dx +\lim_{k \to\infty} \frac{1}{q^{2}} \int_{\varOmega} \vert u_{k} \vert ^{q}\,dx \\ ={}&\liminf_{k \to\infty} \biggl(\frac{1}{2} \int_{\varOmega} \vert \Delta u_{k} \vert ^{2}\,dx- \frac{1}{q} \int_{\varOmega} \vert u_{k} \vert ^{q} \log \vert u_{k} \vert \,dx +\frac{1}{q^{2}} \int_{\varOmega} \vert u_{k} \vert ^{q}\,dx \biggr) \\ ={}&\liminf_{k \to\infty}J(u_{k})=d. \end{aligned}$$

Using (2.2), (2.8), (2.10) and $u_{k}\in\mathscr{N}$, we have

2.12 $$\begin{aligned} I(u)={}& \int_{\varOmega} \vert \Delta u \vert ^{2}\,dx- \int_{\varOmega} \vert u \vert ^{q}\log \vert u \vert \,dx \\ \leq{}&\liminf_{k \to\infty} \int_{\varOmega} \vert \Delta u_{k} \vert ^{2}\,dx-\lim_{k \to \infty} \int_{\varOmega} \vert u_{k} \vert ^{q} \log \vert u_{k} \vert \,dx \\ ={}&\liminf_{k \to\infty} \biggl( \int_{\varOmega} \vert \Delta u_{k} \vert ^{2}\,dx- \int _{\varOmega} \vert u_{k} \vert ^{q} \log \vert u_{k} \vert \,dx \biggr) \\ ={}&\liminf_{k \to\infty}I(u_{k})=0. \end{aligned}$$

By $u_{k}\in\mathscr{N}$ and using the Sobolev embedding inequality and the Poincaré inequality, we have

2.13 $$\begin{aligned} \int_{\varOmega} \vert \Delta u_{k} \vert ^{2}\,dx={}& \int_{\varOmega} \vert u_{k} \vert ^{q}\log \vert u_{k} \vert \,dx \\ \leq{}&\frac{e^{-1}}{\mu_{1}} \int_{\varOmega} \vert u_{k} \vert ^{q+\mu_{1}}\,dx \\ \leq{}&C_{6}^{q+\mu_{1}}\frac{e^{-1}}{ \mu_{1}} \biggl( \int_{\varOmega} \vert Du_{k} \vert ^{2}\,dx \biggr)^{\frac{q+\mu_{1}}{2}} \\ \leq{}&C_{6}^{q+\mu_{1}}C_{7}^{\frac{q+\mu_{1}}{2}} \frac{e^{-1}}{ \mu_{1}} \biggl( \int_{\varOmega} \vert \Delta u_{k} \vert ^{2}\,dx \biggr)^{\frac{q+\mu_{1}}{2}}, \end{aligned}$$

where $C_{6}$ is the Sobolev embedding constant, $C_{7}$ is the Poincaré constant.

By (2.13), we have, for some positive constant $C_{8}$,

2.14 $$\begin{aligned} \int_{\varOmega} \vert u_{k} \vert ^{q} \log \vert u_{k} \vert \,dx= \int_{\varOmega} \vert \Delta u_{k} \vert ^{2}\,dx \geq C_{8}. \end{aligned}$$

Using (2.8) and (2.14), we have

$$\begin{aligned} \frac{e^{-1}}{\mu_{1}} \int_{\varOmega} \vert u \vert ^{q+\mu_{1}}\,dx\geq \int_{\varOmega} \vert u \vert ^{q}\log \vert u \vert \,dx=\lim_{k \to\infty} \int_{\varOmega} \vert u_{k} \vert ^{q}\log \vert u_{k} \vert \,dx\geq C_{8}, \end{aligned}$$

which indicates $u\neq0$. Next, we will study $I(u)=0$. If $I(u)<0$, by Lemma 2.1, there exists a $\overline{\lambda}_{1}$ such that $I(\overline{\lambda}_{1}u)=0$ and $0<\overline{\lambda}_{1}<1$. Thus, $\overline{\lambda}_{1}u\in\mathscr{N}$. By (2.3), (2.4), (2.9) and (2.10), we have

$$\begin{aligned} d\leq{}&J(\overline{\lambda}_{1}u) \\ ={}&\frac{1}{q}I(\overline{ \lambda}_{1}u) + \biggl( \frac{1}{2}-\frac{1}{q} \biggr) \int_{\varOmega}\bigl\vert \Delta(\overline{\lambda}_{1}u) \bigr\vert ^{2} \,dx+\frac{1}{q^{2}} \int_{\varOmega} \vert \overline{\lambda}_{1}u \vert ^{q} \,dx \\ ={}& \biggl(\frac{1}{2}-\frac{1}{q} \biggr) \int_{\varOmega}\bigl\vert \Delta(\overline{\lambda}_{1}u) \bigr\vert ^{2} \,dx+\frac{1}{q^{2}} \int_{\varOmega} \vert \overline{\lambda}_{1}u \vert ^{q} \,dx \\ ={}& \biggl(\frac{1}{2}-\frac{1}{q} \biggr)\overline{ \lambda}_{1}^{2} \int_{\varOmega} \vert \Delta u \vert ^{2}\,dx + \frac{\overline{\lambda}_{1}^{q}}{q^{2}} \int_{\varOmega} \vert u \vert ^{q}\,dx \\ \leq{}& \biggl(\frac{1}{2}-\frac{1}{q} \biggr)\overline{ \lambda}_{1}^{2} \int_{\varOmega} \vert \Delta u \vert ^{2}\,dx+ \frac{\overline{\lambda}_{1}^{2}}{q^{2}} \int_{\varOmega} \vert u \vert ^{q}\,dx \\ ={}&\overline{\lambda}_{1}^{2} \biggl[ \biggl( \frac{1}{2}- \frac{1}{q} \biggr) \int_{\varOmega} \vert \Delta u \vert ^{2}\,dx+ \frac{1}{q^{2}} \int_{\varOmega} \vert u \vert ^{q}\,dx \biggr] \\ \leq{}&\overline{\lambda}_{1}^{2}\liminf _{k \to\infty} \biggl[ \biggl(\frac{1}{2}- \frac{1}{q} \biggr) \int_{\varOmega} \vert \Delta u_{k} \vert ^{2}\,dx+ \frac{1}{q^{2}} \int_{\varOmega} \vert u_{k} \vert ^{q}\,dx \biggr] \\ ={}&\overline{\lambda}_{1}^{2}\liminf_{k \to\infty}J(u_{k}) \\ ={}&\overline{\lambda}_{1}^{2}d, \end{aligned}$$

which indicates $\overline{\lambda}_{1}\geq1$ by $d>0$. It contradicts $0<\overline{\lambda}_{1}<1$. Then, by (2.12), we have $I(u)=0$. Therefore, $u\in\mathscr{N}$. By (2.4), we have $J(u)\geq d$. By (2.11), we have $J(u)\leq d$. So, $J(u)=d$. □

### Lemma 2.3

([9])

*For any* $u\in W_{0}^{1,p}(\varOmega)$, $p\geq1$, *and* $r\geq1$, *the inequality*

$$\begin{aligned} \Vert u \Vert _{q}\leq C \Vert Du \Vert _{p}^{\theta} \Vert u \Vert _{r}^{1-\theta}, \end{aligned}$$

*is valid*, *where*

$$\begin{aligned} \theta= \biggl(\frac{1}{r}-\frac{1}{q} \biggr) \biggl( \frac{1}{n}-\frac{1}{p} +\frac{1}{r} \biggr)^{-1}, \end{aligned}$$

*and for* $p\geq n=1, r\leq q\leq\infty$; *for* $n>1$ *and* $p< n$, $q\in[r,p^{*}]$ *if* $r\leq p^{*}$ *and* $q\in[p^{*},r]$ *if* $r\geq p^{*}$; *for* $p=n>1, r\leq q\leq\infty$; *for* $p>n>1, r\leq q\leq\infty$.

*Here*, *the constant* *C* *depends on* *n*, *p*, *q* *and* *r*.

### Lemma 2.4

([12])

*Let* $h:\mathbb {R}^{+}\rightarrow R^{+}$ *be a nonincreasing function*. *Assume that there is a constant* $A>0$ *such that*

$$\begin{aligned} \int_{s}^{+\infty}h(t)\,dt\leq Ah(s),\quad 0\leq s< +\infty. \end{aligned}$$

*Then* $h(t)\leq h(0)e^{1-\frac{t}{A}}$, *for all* $t>0$.

## Local existence and uniqueness

### Definition 3.1

(Weak solution)

A function *u* is a solution of problem (1.1) over $[0,T]$ if $u\in L^{\infty}(0,T;H_{0}^{2}(\varOmega))$ with $u_{t}\in L^{2}(0,T;L^{2}(\varOmega))$, satisfies the initial condition $u(0)=u_{0}(x)\in H_{0}^{2}(\varOmega)\setminus\{0\}$, and

3.1 $$\begin{aligned} \int_{\varOmega}u_{t}w\,dx+ \int_{\varOmega}\Delta u\Delta w\,dx= \int_{\varOmega} \vert u \vert ^{q-2}u\log \vert u \vert w\,dx, \end{aligned}$$

for any $w\in H_{0}^{2}(\varOmega)$, and for a.e. $t\in[0,T]$.

### Theorem 3.1

(Local existence)

*Let* $u_{0}\in H_{0}^{2}(\varOmega)\setminus\{0\}$. *Then there exists a positive constant* $T_{0}$ *such that the problem* (1.1) *has a unique weak solution* $u(x,t)$ *on* $\varOmega\times(0,T_{0})$. *Furthermore*, $u(x,t)$ *satisfies the energy inequality*

3.2 $$\begin{aligned} \int_{0}^{t} \int_{\varOmega}u_{s}^{2}\,dx\,ds+J \bigl(u(t) \bigr)\leq J(u_{0}),\quad t\in[0,T_{0}]. \end{aligned}$$

### Proof

In the space of $H_{0}^{2}(\varOmega)$, we take a basis $\{w_{j}\} _{j=1}^{\infty}$ and define the finite dimensional space

$$\begin{aligned} V_{m}=\operatorname{span}\{w_{1},w_{2}, \ldots,w_{m}\}. \end{aligned}$$

Let $u_{0m}$ be an element of $V_{m}$ such that

3.3 $$\begin{aligned} u_{0m}=\sum_{j=1}^{m}a_{mj}w_{j} \rightarrow u_{0}\quad \mbox{strongly in }H_{0}^{2}( \varOmega), \end{aligned}$$

as $m\rightarrow\infty$. We can find the approximate solution $u_{m}(x,t)$ of the problem (1.1) in the form

3.4 $$\begin{aligned} u_{m}(x,t)=\sum_{j=1}^{m} \alpha_{mj}(t)w_{j}(x), \end{aligned}$$

where $\alpha_{mj}\ (1\leq j\leq m)$ satisfy the ordinary differential equations

3.5 $$\begin{aligned} \int_{\varOmega}u_{mt}w_{i}\,dx+ \int_{\varOmega}\Delta u_{m}\Delta w_{i}\,dx= \int _{\varOmega} \vert u_{m} \vert ^{q-2}u_{m} \log \vert u_{m} \vert w_{i}\,dx, \end{aligned}$$

for $i\in\{1,2,\ldots,m\}$, with

3.6 $$\begin{aligned} \alpha_{mj}(0)=a_{mj},\quad i\in\{1,2, \ldots,m\}. \end{aligned}$$

We find from Peano’s theorem that (3.5)–(3.6) has a local solution $\alpha_{mj}$, and there exists a positive $T_{m}>0$ such that $\alpha_{mj}\in C^{1}([0,T_{m}])$, therefore $u_{m}\in C^{1}([0,T_{m}];H_{0}^{2}(\varOmega))$. Multiplying the *i*th equation in (3.5) by $\alpha_{mi}$, summing over *i* from 1 to *m*, we have

3.7 $$\begin{aligned} \int_{\varOmega}u_{mt}u_{m}\,dx+ \int_{\varOmega} \vert \Delta u_{m} \vert ^{2}\,dx= \int_{\varOmega} \vert u_{m} \vert ^{q} \log \vert u_{m} \vert \,dx. \end{aligned}$$

Integrating the above formula with respect to *s* over $(0,t)$, we have

3.8 $$\begin{aligned} y_{m}(t)=y_{m}(0)+ \int_{0}^{t} \int_{\varOmega} \vert u_{m} \vert ^{q}\log \vert u_{m} \vert \,dx\,ds, \end{aligned}$$

where

3.9 $$\begin{aligned} y_{m}(t)=\frac{1}{2} \int_{\varOmega} \vert u_{m} \vert ^{2}\,dx+ \int_{0}^{t} \int_{\varOmega} \vert \Delta u_{m} \vert ^{2}\,dx\,ds. \end{aligned}$$

Choose $\mu_{2}$ such that $0<\mu_{2}<2+\frac{4}{n}-q$. Using Lemma 2.3, the Poincaré inequality and the Young inequality, we have

3.10 $$\begin{aligned} & \int_{\varOmega} \vert u_{m} \vert ^{q}\log \vert u_{m} \vert \,dx \\ &\quad \leq\frac{e^{-1}}{\mu_{2}} \int_{\varOmega} \vert u_{m} \vert ^{q+\mu_{2}}\,dx \\ &\quad \leq\frac{e^{-1}}{\mu_{2}}C_{9}^{q+\mu_{2}} \Vert Du_{m} \Vert _{2}^{\theta(q+\mu_{2})} \Vert u_{m} \Vert _{2}^{(1-\theta)(q+\mu_{2})} \\ &\quad \leq\frac{e^{-1}}{\mu_{2}}C_{9}^{q+\mu_{2}}C_{10}^{\frac{\theta(q+\mu _{2})}{2}} \Vert \Delta u_{m} \Vert _{2}^{\theta(q+\mu_{2})} \Vert u_{m} \Vert _{2}^{(1-\theta)(q+\mu_{2})} \\ &\quad \leq\varepsilon \Vert \Delta u_{m} \Vert _{2}^{2}+ \biggl( \frac{\varepsilon\mu_{2}}{e^{-1}C_{9}^{q+\mu_{2}}C_{10}^{\frac{\theta(q+\mu_{2})}{2}}} \biggr)^{-\frac{\theta(q+\mu _{2})}{2-\theta(q+\mu_{2})}} \Vert u_{m} \Vert _{2}^{\frac{2(1-\theta)(q+\mu_{2})}{2-\theta (q+\mu_{2})}}, \end{aligned}$$

where $C_{9}$ is the constant of Lemma 2.3, $C_{10}$ is the Poincaré constant, $0<\varepsilon<1$, and $\theta=n(\frac {1}{2}-\frac{1}{q+\mu_{2}})$. Let $\gamma=\frac{(1-\theta)(q+\mu_{2})}{2-\theta(q+\mu_{2})}$ and $C_{11}= (\frac{\varepsilon\mu_{2}}{e^{-1}C_{9}^{q+\mu_{2}}C_{10}^{\frac{\theta(q+\mu_{2})}{2}}} ) ^{-\frac{\theta(q+\mu_{2})}{2-\theta(q+\mu_{2})}}$, thus (3.10) becomes

3.11 $$\begin{aligned} \int_{\varOmega} \vert u_{m} \vert ^{q}\log \vert u_{m} \vert \,dx\leq&\varepsilon \int_{\varOmega} \vert \Delta u_{m} \vert ^{2}\,dx +C_{11} \biggl( \int_{\varOmega} \vert u_{m} \vert ^{2}\,dx \biggr)^{\gamma}. \end{aligned}$$

It is easy to check $\gamma>1$ according to $2< q<2+\frac{4}{n}$. Using (3.3), (3.8), (3.9) and (3.11), we have

3.12 $$\begin{aligned} y_{m}(t)={}&y_{m}(0)+ \int_{0}^{t} \int_{\varOmega} \vert u_{m} \vert ^{q}\log \vert u_{m} \vert \,dx\,ds \\ \leq{}&y_{m}(0)+ \int_{0}^{t} \biggl[\varepsilon \int_{\varOmega} \vert \Delta u_{m} \vert ^{2} \,dx+C_{11} \biggl( \int_{\varOmega} \vert u_{m} \vert ^{2}\,dx \biggr) ^{\gamma} \biggr]\,ds \\ ={}&\frac{1}{2} \int_{\varOmega}\bigl\vert u_{m}(0) \bigr\vert ^{2}\,dx+ \int_{0}^{t} \int_{\varOmega}\bigl\vert \Delta u_{m}(0) \bigr\vert ^{2}\,dx\,ds \\ &{}+ \int_{0}^{t} \biggl[\varepsilon \int_{\varOmega} \vert \Delta u_{m} \vert ^{2}\,dx +C_{11} \biggl( \int_{\varOmega} \vert u_{m} \vert ^{2}\,dx \biggr) ^{\gamma} \biggr]\,ds \\ \leq{}&C_{12}+ \int_{0}^{t} \biggl[\varepsilon \int_{\varOmega} \vert \Delta u_{m} \vert ^{2}\,dx +C_{11} \biggl( \int_{\varOmega} \vert u_{m} \vert ^{2}\,dx \biggr) ^{\gamma} \biggr]\,ds \\ \leq{}&C_{12}+\varepsilon \int_{0}^{t} \int_{\varOmega} \vert \Delta u_{m} \vert ^{2}\,dx\,ds +C_{11} \int_{0}^{t} \biggl( \int_{\varOmega} \vert u_{m} \vert ^{2}\,dx \biggr) ^{\gamma}\,ds \\ \leq{}&C_{12}+\frac{\varepsilon}{2} \int_{\varOmega} \vert u_{m} \vert ^{2}\,dx+ \varepsilon \int _{0}^{t} \int_{\varOmega} \vert \Delta u_{m} \vert ^{2}\,dx\,ds \\ &{}+C_{11}2^{\gamma} \int_{0}^{t} \biggl( \int_{0}^{s} \int_{\varOmega} \vert \Delta u_{m} \vert ^{2}\,dx\,dy \biggr)^{\gamma}\,ds \\ &{}+C_{11}2^{\gamma} \int_{0}^{t} \biggl(\frac{1}{2} \int_{\varOmega} \vert u_{m} \vert ^{2}\,dx \biggr) ^{\gamma}\,ds \\ \leq{}&C_{12}+\varepsilon y_{m}(t)+C_{11}2^{\gamma} \int_{0}^{t}y_{m}(s)^{\gamma}\,ds. \end{aligned}$$

Using $0<\varepsilon<1$ and (3.12),

3.13 $$\begin{aligned} y_{m}(t)\leq\frac{C_{12}}{1-\varepsilon}+\frac{C_{11}2^{\gamma}}{ 1-\varepsilon} \int_{0}^{t}y_{m}(s)^{\gamma}\,ds. \end{aligned}$$

Using the integral inequality of Gronwall–Bellman–Bihari type and combining with (3.13), there exists $T_{0}$ such that

3.14 $$\begin{aligned} y_{m}(t)\leq C_{13}(T_{0}),\quad t \in[0,T_{0}], \end{aligned}$$

where $C_{13}(T_{0})$ is a positive constant dependent on $T_{0}$. Multiplying equation (3.5) by $\alpha'_{mi}$, summing over *i* from 1 to *m* and integrating with respect to time variable on $[0,t]$, we have

3.15 $$\begin{aligned} \int_{0}^{t} \int_{\varOmega}u_{ms}^{2}\,dx\,ds+J \bigl(u_{m}(t) \bigr)=J \bigl(u_{m}(0) \bigr),\quad \mbox{for all }t \in[0,T_{0}]. \end{aligned}$$

We find from (3.3) and the continuity of the *J* that there exists a constant $C_{14}>0$ such that

3.16 $$\begin{aligned} J \bigl(u_{m}(0) \bigr)\leq C_{14},\quad \mbox{for all } m. \end{aligned}$$

Using (2.1), (3.9), (3.11), (3.14), (3.15) and (3.16), we have

3.17 $$\begin{aligned} C_{14}\geq{}& J \bigl(u_{m}(t) \bigr) \\ ={}&\frac{1}{2} \int_{\varOmega}\bigl\vert \Delta u_{m}(t) \bigr\vert ^{2}\,dx- \frac{1}{q} \int_{\varOmega}\bigl\vert u_{m}(t) \bigr\vert ^{q} \log \bigl\vert u_{m}(t) \bigr\vert \,dx + \frac{1}{q^{2}} \int_{\varOmega}\bigl\vert u_{m}(t) \bigr\vert ^{q}\,dx \\ \geq{}&\frac{1}{2} \int_{\varOmega}\bigl\vert \Delta u_{m}(t) \bigr\vert ^{2}\,dx- \frac{1}{q} \int _{\varOmega}\bigl\vert u_{m}(t) \bigr\vert ^{q} \log \bigl\vert u_{m}(t) \bigr\vert \,dx \\ \geq{}&\frac{1}{2} \int_{\varOmega}\bigl\vert \Delta u_{m}(t) \bigr\vert ^{2}\,dx- \frac{\varepsilon }{q} \int_{\varOmega}\bigl\vert \Delta u_{m}(t) \bigr\vert ^{2}\,dx - \frac{C_{11}}{q} \biggl( \int_{\varOmega}\bigl\vert u_{m}(t) \bigr\vert ^{2}\,dx \biggr)^{\gamma} \\ ={}& \biggl(\frac{1}{2}-\frac{\varepsilon}{q} \biggr) \int_{\varOmega}\bigl\vert \Delta u_{m}(t) \bigr\vert ^{2}\,dx - \frac{C_{11}}{q} \biggl( \int_{\varOmega}\bigl\vert u_{m}(t) \bigr\vert ^{2}\,dx \biggr)^{\gamma} \\ \geq{}& \biggl(\frac{1}{2}-\frac{\varepsilon}{q} \biggr) \int_{\varOmega}\bigl\vert \Delta u_{m}(t) \bigr\vert ^{2}\,dx - \frac{C_{11}}{q} \bigl(2C_{13}(T_{0}) \bigr)^{\gamma}, \end{aligned}$$

which implies that

3.18 $$\begin{aligned} \int_{\varOmega}\bigl\vert \Delta u_{m}(t) \bigr\vert ^{2}\,dx\leq \biggl(\frac{1}{2}-\frac {\varepsilon}{q} \biggr)^{-1} \biggl[C_{14}+\frac{C_{11}}{ q} \bigl(2C_{13}(T_{0}) \bigr)^{\gamma} \biggr]. \end{aligned}$$

By the Poincaré inequality and (3.18), we obtain

3.19 $$\begin{aligned} \int_{\varOmega}\bigl\vert u_{m}(t) \bigr\vert ^{2}\,dx\leq{}& C_{15} \int_{\varOmega}\bigl\vert Du_{m}(t) \bigr\vert ^{2}\,dx\leq C_{15}C_{16} \int_{\varOmega}\bigl\vert \Delta u_{m}(t) \bigr\vert ^{2}\,dx \\ \leq{}& C_{15}C_{16} \biggl(\frac{1}{2}- \frac{\varepsilon}{q} \biggr)^{-1} \biggl[C_{14}+ \frac{C_{11}}{ q} \bigl(2C_{13}(T_{0}) \bigr)^{\gamma} \biggr], \end{aligned}$$

where $C_{15},C_{16}$ are the Poincaré constants. We can easily obtain from the above inequality

3.20 $$\begin{aligned} \Vert u_{m} \Vert _{L^{\infty}(0,T_{0};H_{0}^{2}(\varOmega))}\leq C_{17}(T_{0}), \end{aligned}$$

where $C_{17}(T_{0})$ is a positive constant dependent on $T_{0}$. Using (3.15)–(3.17), we have

3.21 $$\begin{aligned} \biggl(\frac{1}{2}-\frac{\varepsilon}{q} \biggr) \int_{\varOmega}\bigl\vert \Delta u_{m}(t) \bigr\vert ^{2}\,dx - \frac{C_{11}}{q} \bigl(2C_{13}(T_{0}) \bigr)^{\gamma}+ \int_{0}^{t} \int _{\varOmega}u_{ms}^{2}\,dx\,ds\leq C_{14}, \end{aligned}$$

which implies that

3.22 $$\begin{aligned} \Vert u_{mt} \Vert _{L^{2}(0,T_{0};L^{2}(\varOmega))}\leq C_{18}(T_{0}), \end{aligned}$$

where $C_{18}(T_{0})$ is a positive constant dependent on $T_{0}$. It follows from (3.20) and (3.22) that there exist a function *u* and a subsequence of $\{u_{m}\}_{m=1}^{\infty}$ still denoted $\{u_{m}\}_{m=1}^{\infty}$ such that

3.23 $$\begin{aligned} &u_{m}\rightarrow u\quad \text{weakly star in } {L^{\infty} \bigl(0,T_{0};H_{0}^{2}( \varOmega) \bigr)}, \end{aligned}$$

3.24 $$\begin{aligned} & u_{mt}\rightarrow u_{t} \quad\text{weakly in } {L^{2} \bigl(0,T_{0};L^{2}(\varOmega) \bigr)}. \end{aligned}$$

We obtain from the Aubin–Lions–Simon lemma (see [13]) together with (3.23) and (3.24)

3.25 $$\begin{aligned} u_{m}\rightarrow u\quad \mbox{strongly in }C \bigl(0,T_{0};L^{2}( \varOmega) \bigr). \end{aligned}$$

So, $u_{m}\rightarrow u$ a.e. $(x,t)\in\varOmega\times(0,T_{0})$. This implies that

3.26 $$\begin{aligned} \vert u_{m} \vert ^{q-2}u_{m} \log \vert u_{m} \vert \rightarrow \vert u \vert ^{q-2}u\log \vert u \vert \quad \mbox{a.e. }(x,t)\in \varOmega \times(0,T_{0}). \end{aligned}$$

According to $2< q<2+\frac{4}{n}$, we can choose $\mu_{3}$ such that $1<\frac{q(q-1+\mu_{3})}{q-1}<\frac{2n}{n-2}$. Then, using the Sobolev embedding inequality and combining (3.19), we have

3.27 $$\begin{aligned} & \int_{\varOmega}\bigl\vert \vert u_{m} \vert ^{q-2}u_{m} \log \vert u_{m} \vert \bigr\vert ^{\frac{q}{q-1}}\,dx \\ &\quad= \int_{\{x\in\varOmega: \vert u_{m} \vert \leq1\}} \bigl\vert \vert u_{m} \vert ^{q-2}u_{m} \log \vert u_{m} \vert \bigr\vert ^{\frac{q}{q-1}}\,dx \\ &\qquad{}+ \int_{\{x\in\varOmega: \vert u_{m} \vert \geq1\}} \bigl\vert \vert u_{m} \vert ^{q-2}u_{m} \log \vert u_{m} \vert \bigr\vert ^{\frac{q}{q-1}}\,dx \\ &\quad \leq \bigl(e(q-1) \bigr)^{-\frac{q}{q-1}} \vert \varOmega \vert + \biggl(\frac{e^{-1}}{\mu _{3}} \biggr) ^{-\frac{q}{q-1}} \int_{\varOmega} \vert u_{m} \vert ^{\frac{q(q-1+\mu_{3})}{q-1}}\,dx \\ &\quad \leq \bigl(e(q-1) \bigr)^{-\frac{q}{q-1}} \vert \varOmega \vert + \biggl(\frac{e^{-1}}{\mu _{3}} \biggr) ^{-\frac{q}{q-1}}C_{19}^{{\frac{q(q-1+\mu_{3})}{q-1}}} \biggl( \int_{\varOmega} \vert Du_{m} \vert ^{2}\,dx \biggr)^{{\frac{q(q-1+\mu_{3})}{2(q-1)}}} \\ &\quad \leq \bigl(e(q-1) \bigr)^{-\frac{q}{q-1}} \vert \varOmega \vert + \biggl(\frac{e^{-1}}{\mu _{3}} \biggr) ^{-\frac{q}{q-1}}C_{19}^{{\frac{q(q-1+\mu_{3})}{q-1}}} \\ &\qquad{}\times \biggl(C_{16} \biggl(\frac{1}{2}- \frac{\varepsilon}{q} \biggr)^{-1} \biggl[C_{14}+ \frac{C_{11}}{ q} \bigl(2C_{13}(T_{0}) \bigr)^{\gamma} \biggr] \biggr)^{{\frac{q(q-1+\mu_{3})}{2(q-1)}}}, \end{aligned}$$

where $C_{19}$ is the embedding constant. Using (3.26), (3.27) and Lion’s lemma (see [13]), we obtain

3.28 $$\begin{aligned} \vert u_{m} \vert ^{q-2}u_{m} \log \vert u_{m} \vert \rightarrow \vert u \vert ^{q-2}u\log \vert u \vert \quad\text{weakly$^{*}$ in } {L^{\infty} \bigl(0,T_{0};L^{\frac{q}{q-1}}(\varOmega) \bigr)}. \end{aligned}$$

Passing to the limit in (3.5) and (3.6) as $m\rightarrow \infty$, by (3.23), (3.24) and (3.28), we see that *u* satisfies the initial condition $u(0)=u_{0}$ and

3.29 $$\begin{aligned} \int_{\varOmega}u_{t}(t)w\,dx+ \int_{\varOmega}\Delta u(t)\Delta w\,dx= \int _{\varOmega}\bigl\vert u(t) \bigr\vert ^{q-2}u(t) \log \bigl\vert u(t) \bigr\vert w\,dx, \end{aligned}$$

for all $w\in H_{0}^{2}(\varOmega)$, and for a.e. $t\in[0,T_{0}]$. So, *u* is a desired solution of problem (1.1).

Next, we will study uniqueness of the solution. We obtain from (3.29) for any $v\in L^{2}(0,T_{0};H_{0}^{2}(\varOmega))$

3.30 $$\begin{aligned} \int_{\varOmega}u_{t}(t)v\,dx+ \int_{\varOmega}\Delta u(t)\Delta v\,dx= \int _{\varOmega}\bigl\vert u(t) \bigr\vert ^{q-2}u(t) \log \bigl\vert u(t) \bigr\vert v\,dx. \end{aligned}$$

We suppose there are two solutions $u_{1}$ and $u_{2}$. Let $w=u_{1}-u_{2}$, thus we have $w(0)=0$, $w\in L^{2}(0,T_{0};H_{0}^{2}(\varOmega))$ and $w_{t}\in L^{2}(0,T_{0};L^{2}(\varOmega))$. We set

$$\begin{aligned} v(s)= \textstyle\begin{cases} u_{1}(s)-u_{2}(s),& s\in[0,t], \\ 0, &s\in[t,T_{0}]. \end{cases}\displaystyle \end{aligned}$$

From (3.30), it follows that

3.31 $$\begin{aligned} & \int_{0}^{t} \int_{\varOmega}w_{s}w\,dx\,ds+ \int_{0}^{t} \int_{\varOmega} \vert \Delta w \vert ^{2}\,dx\,ds \\ &\quad = \int_{0}^{t} \int_{\varOmega}\bigl( \vert u_{1} \vert ^{q-2}u_{1} \log \vert u_{1} \vert - \vert u_{2} \vert ^{q-2}u_{2} \log \vert u_{2} \vert \bigr)w\,dx\,ds. \end{aligned}$$

According to $0\leq\int_{0}^{t}\int_{\varOmega}|\Delta w|^{2}\,dx\,ds$, (3.31) becomes

3.32 $$\begin{aligned} \int_{0}^{t} \int_{\varOmega}w_{s}w\,dx\,ds \leq \int_{0}^{t} \int_{\varOmega}\bigl( \vert u_{1} \vert ^{q-2}u_{1} \log \vert u_{1} \vert - \vert u_{2} \vert ^{q-2}u_{2} \log \vert u_{2} \vert \bigr)w\,dx\,ds. \end{aligned}$$

We construct a function $f:{\mathbb {R}}^{*}\rightarrow\mathbb {R}$, $f(s)=|s|^{q-2}s\log|s|$. Thus, we find that there exists a positive constant $C_{20}$ such that

3.33 $$\begin{aligned} \bigl\vert \vert u_{1} \vert ^{q-2}u_{1} \log \vert u_{1} \vert - \vert u_{2} \vert ^{q-2}u_{2} \log \vert u_{2} \vert \bigr\vert \leq C_{20} \vert w \vert . \end{aligned}$$

By (3.32) and (3.33),

$$\begin{aligned} \int_{0}^{t} \int_{\varOmega}w_{s}w\,dx\,ds \leq C_{20} \int_{0}^{t} \int_{\varOmega}w^{2}\,dx\,ds, \end{aligned}$$

i.e.,

3.34 $$\begin{aligned} \frac{1}{2} \int_{\varOmega}w^{2}\,dx \leq\frac{1}{2} \int_{\varOmega}w(0)^{2}\,dx+C_{20} \int_{0}^{t} \int_{\varOmega}w^{2}\,dx\,ds \leq C_{20} \int_{0}^{t} \int_{\varOmega}w^{2}\,dx\,ds. \end{aligned}$$

Using Gronwall’s inequality and combining with (3.34), we have

$$\begin{aligned} \int_{\varOmega}w^{2}\,dx\leq0. \end{aligned}$$

So, the uniqueness is derived.

Finally, we will study (3.2). Let $\phi(t)$ is a nonnegative function which belongs to $C([0,T_{0}])$. From (3.15), we can obtain

3.35 $$\begin{aligned} & \int_{0}^{T_{0}}\phi(t)\,dt \int_{0}^{t} \int_{\varOmega}u_{ms}^{2}\,dx\,ds+ \int _{0}^{T_{0}}J \bigl(u_{m}(t) \bigr) \phi(t) \,dt \\ &\quad = \int_{0}^{T_{0}}J \bigl(u_{m}(0) \bigr) \phi(t) \,dt. \end{aligned}$$

As $m\rightarrow\infty$,

$$\int_{0}^{T_{0}}J \bigl(u_{m}(0) \bigr) \phi(t) \,dt\rightarrow \int_{0}^{T_{0}}J(u_{0})\phi(t)\,dt $$

and

$$\int_{0}^{T_{0}}\phi(t)\,dt \int_{0}^{t} \int_{\varOmega}u_{ms}^{2}\,dx\,ds\rightarrow \int _{0}^{T_{0}}\phi(t)\,dt \int_{0}^{t} \int_{\varOmega}u_{s}^{2}\,dx\,ds $$

hold. Since $\int_{0}^{T_{0}}J(u_{m}(t))\phi(t)\,dt$ is lower semi-continuous with respect to the weak topology of $L^{2}(0,T_{0};H_{0}^{2}(\varOmega))$, we know that

$$\int_{0}^{T_{0}}J \bigl(u(t) \bigr)\phi(t)\,dt\leq \liminf_{m \to\infty} \int _{0}^{T_{0}}J \bigl(u_{m}(t) \bigr) \phi(t) \,dt. $$

Hence, by (3.35), it follows that

$$\begin{aligned} \int_{0}^{T_{0}}\phi(t)\,dt \int_{0}^{t} \int_{\varOmega}u_{s}^{2}\,dx\,ds+ \int _{0}^{T_{0}}J \bigl(u(t) \bigr)\phi(t)\,dt\leq \int_{0}^{T_{0}}J(u_{0})\phi(t)\,dt, \end{aligned}$$

as $m\rightarrow\infty$. $\phi(t)$ is arbitrary nonnegative function, so we have

$$\begin{aligned} \int_{0}^{t} \int_{\varOmega}u_{s}^{2}\,dx\,ds+J \bigl(u(t) \bigr)\leq J(u_{0}),\quad t\in[0,T_{0}]. \end{aligned}$$

 □

## Global existence and decay estimates

Now as in [9], we introduce the following sets: $\mathscr{W}_{1}=\{u\in H_{0}^{2}(\varOmega)\setminus\{0\}:J(u)< d\}$, $\mathscr{W}_{2}=\{u\in H_{0}^{2}(\varOmega)\setminus\{0\}:J(u)=d\}$, $\mathscr{W}_{1}^{+}=\{u\in\mathscr{W}_{1}:I(u)>0\}$, $\mathscr{W}_{2}^{+}=\{u\in\mathscr{W}_{2}:I(u)>0\}$, $\mathscr{W}_{1}^{-}=\{u\in\mathscr{W}_{1}:I(u)<0\}$, $\mathscr{W}_{2}^{-}=\{u\in\mathscr{W}_{2}:I(u)<0\}$, $\mathscr{W}=\mathscr{W}_{1}\cup\mathscr{W}_{2}$, $\mathscr{W}^{+}=\mathscr{W}_{1}^{+}\cup\mathscr{W}_{2}^{+}$, $\mathscr{W}^{-}=\mathscr{W}_{1}^{-}\cup\mathscr{W}_{2}^{-}$.

### Definition 4.1

(Maximal existence time)

Let $u(t)$ be a solution of problem (1.1). We define the maximal existence time $T_{\mathrm{max}}$ as follows:

$$\begin{aligned} T_{\mathrm{max}}=\sup \bigl\{ T>0:u(t)\mbox{ exists on }[0,T] \bigr\} . \end{aligned}$$

Then:

(i) if $T_{\mathrm{max}}<{+\infty}$, we say that $u(t)$ blows up in finite time and $T_{\mathrm{max}}$ is the blow-up time;
(ii) if $T_{\mathrm{max}}={+\infty}$, we say that $u(t)$ is global.

### Theorem 4.1

*Let* $u_{0}\in\mathscr{W^{+}}$. *Then the problem of* (1.1) *admits a unique global weak solution such that*

$$\begin{aligned} u(t)\in\overline{\mathscr{W^{+}}},\quad t\in[0,\infty), \end{aligned}$$

*and*

4.1 $$\begin{aligned} \int_{0}^{t} \int_{\varOmega}u_{s}^{2}\,dx\,ds+J \bigl(u(t) \bigr)\leq J(u_{0}),\quad t\in[0,\infty). \end{aligned}$$

*Furthermore*, *if* $u_{0}\in\mathscr{W}_{1}^{+}$, *the solution* $u(t)$ *decays exponentially*.

### Proof

We will consider the following two cases.

First we address the case of the initial data $u_{0}\in\mathscr{W}_{1}^{+}$.

Let $\{w_{j}\}_{j=1}^{\infty}$, $\{u_{0m}\}_{m=1}^{\infty}$, and $\{u_{m}\} _{m=1}^{\infty}$ be the same as those stated in the proof of the local existence in the second section. Multiplying the (3.5) by $\alpha '_{mi}(t)$, summing over *i* from 1 to *m* and integrating with respect to time variable on $[0,t]$, we have

4.2 $$\begin{aligned} \int_{0}^{t} \int_{\varOmega}u_{ms}^{2}\,dx\,ds+J \bigl(u_{m}(t) \bigr)=J \bigl(u_{m}(0) \bigr), \quad t \in[0,T_{\mathrm{max}}), \end{aligned}$$

where $T_{\mathrm{max}}$ is the maximal existence time of solution $u_{m}(x,t)$. We will prove that $T_{\mathrm{max}}=\infty$.

By (3.3), (3.6) and the continuity of *J*, we have

4.3 $$\begin{aligned} J \bigl(u_{m}(0) \bigr)\rightarrow J(u_{0}) \quad \mbox{as }m\rightarrow\infty. \end{aligned}$$

Using (4.2) and (4.3) and combining with $J(u_{0})< d$, we have

4.4 $$\begin{aligned} \int_{0}^{t} \int_{\varOmega}u_{ms}^{2}\,dx\,ds+J \bigl(u_{m}(t) \bigr)< d,\quad t\in[0,T_{\mathrm{max}}), \end{aligned}$$

for sufficiently large *m*. Next, we will study

4.5 $$\begin{aligned} u_{m}(t)\in\mathscr{W}_{1}^{+}, \quad t \in[0,T_{\mathrm{max}}), \end{aligned}$$

for sufficiently large *m*. We assume that (4.5)  does not hold and think that there exists a smallest time $t_{0}$ such that $u_{m}(t_{0})\notin\mathscr{W}_{1}^{+}$. Then, we have $u_{m}(t_{0})\in\partial {\mathscr{W}_{1}^{+}}$. So, we have

4.6 $$\begin{aligned} J \bigl(u_{m}(t_{0}) \bigr)=d, \end{aligned}$$

or

4.7 $$\begin{aligned} I \bigl(u_{m}(t_{0}) \bigr)=0. \end{aligned}$$

(4.6) contradicts with (4.4). If (4.7) holds, from (2.4) we can obtain

$$\begin{aligned} J \bigl(u_{m}(t_{0}) \bigr)\geq\inf_{u\in\mathscr{N}}J(u)=d, \end{aligned}$$

which contradicts with (4.4). Hence, we have (4.5), i.e., $J(u_{m}(t))< d$, and $I(u_{m}(t))>0$, for any $t\in[0,T_{\mathrm{max}})$, for sufficiently large *m*. Then, by (2.3), we have

4.8 $$\begin{aligned} d>J \bigl(u_{m}(t) \bigr)={}&\frac{1}{q}I \bigl(u_{m}(t) \bigr)+ \biggl(\frac{1}{2}-\frac{1}{q} \biggr) \int_{\varOmega}\bigl\vert \Delta u_{m}(t) \bigr\vert ^{2}\,dx+ \frac{1}{q^{2}} \int_{\varOmega}\bigl\vert u_{m}(t) \bigr\vert ^{q}\,dx \\ \geq{}& \biggl(\frac{1}{2}-\frac{1}{q} \biggr) \int_{\varOmega}\bigl\vert \Delta u_{m}(t) \bigr\vert ^{2}\,dx+ \frac{1}{q^{2}} \int_{\varOmega}\bigl\vert u_{m}(t) \bigr\vert ^{q}\,dx \\ \geq{}& \biggl(\frac{1}{2}-\frac{1}{q} \biggr) \int_{\varOmega}\bigl\vert \Delta u_{m}(t) \bigr\vert ^{2}\,dx. \end{aligned}$$

Using (4.8) and combining with the Poincaré inequality, we have

4.9 $$\begin{aligned} \int_{\varOmega}\bigl\vert u_{m}(t) \bigr\vert ^{2}\,dx&\leq C_{21} \int_{\varOmega}\bigl\vert D u_{m}(t) \bigr\vert ^{2}\,dx\leq C_{21}C_{22} \int_{\varOmega}\bigl\vert \Delta u_{m}(t) \bigr\vert ^{2}\,dx \\ &\leq \biggl(\frac{1}{2}-\frac{1}{q} \biggr)^{-1}C_{21}C_{22} d, \end{aligned}$$

where $C_{21}$ and $C_{22}$ are the Poincaré constants. By (4.4) and (4.8), we have

4.10 $$\begin{aligned} \int_{0}^{t} \int_{\varOmega}u_{ms}^{2}\,dx\,ds+ \biggl( \frac{1}{2}-\frac{1}{q} \biggr) \int_{\varOmega}\bigl\vert \Delta u_{m}(t) \bigr\vert ^{2}\,dx< d. \end{aligned}$$

Equations (4.9) and (4.10) imply that $T_{\mathrm{max}}=\infty$. Then the rest is similar to the proof of the local existence, and we see that there exists a unique global weak solution $u(t)\in\mathscr {W}_{1}^{+}$ of the problem (1.1), and

$$\begin{aligned} \int_{0}^{t} \int_{\varOmega}u_{s}^{2}\,dx\,ds+J \bigl(u(t) \bigr)\leq J(u_{0}), \quad t\in[0,\infty). \end{aligned}$$

Now we address the case of the initial data $u_{0}\in\mathscr{W}_{2}^{+}$.

First, we can choose a sequence $\{\rho_{m}\}_{m=1}^{\infty}\subset(0,1)$ and $\lim_{m \to\infty}\rho_{m}=1$. Next, we consider the following problem:

4.11 $$\begin{aligned} \textstyle\begin{cases} u_{t}+\Delta^{2}u= \vert u \vert ^{q-2}u\log \vert u \vert ,\quad x\in\varOmega, t>0, \\ u(x,t)=\Delta u(x,t)=0,\quad x\in\partial\varOmega, t>0, \\ u(x,0)=u_{0m}(x),\quad x\in\varOmega, \end{cases}\displaystyle \end{aligned}$$

where $u_{0m}=\rho_{m}u_{0}$. By $I(u_{0})>0$ and Lemma 2.1, we see that there exists a $\overline{\lambda}_{2}>1$. Hence, $I(u_{0m})=I(\rho _{m}u_{0})>0$ and $J(u_{0m})=J(\rho_{m}u_{0})< J(u_{0})=d$ hold. So, we have $u_{0m}\in\mathscr{W}_{1}^{+}$. Similar to the previous case, we see that the problem (4.11) admits that, for any positive *m*, there exists a unique global $u_{m}$ which satisfies $u_{m}\in L^{\infty}(0,\infty;H_{0}^{2}(\varOmega))$, $u_{mt}\in L^{2}(0,\infty ;L^{2}(\varOmega))$, $u_{m}(0)=u_{0m}=\rho_{m}u_{0}\rightarrow u_{0}$ strongly in $H_{0}^{2}(\varOmega)$, and

4.12 $$\begin{aligned} \int_{\varOmega}u_{mt}w\,dx+ \int_{\varOmega}\Delta u_{m}\Delta w\,dx= \int _{\varOmega} \vert u_{m} \vert ^{q-2}u_{m} \log \vert u_{m} \vert w\,dx, \end{aligned}$$

for any $w\in H_{0}^{2}(\varOmega)$, and for a.e. $t\in[0,\infty)$. Moreover, we have

$$\begin{aligned} u_{m}(t)\in\mathscr{W}_{1}^{+}, \quad t\in[0, \infty) \end{aligned}$$

and

$$\begin{aligned} \int_{0}^{t} \int_{\varOmega}u_{ms}^{2}\,dx\,ds+J \bigl(u_{m}(t) \bigr)\leq J(u_{0m})< d, \quad t\in [0,\infty). \end{aligned}$$

The remainder of the proof can be processed as the previous case.

Finally, we discuss the decay results.

Since $u_{0}\in\mathscr{W}_{1}^{+}$, similar to the first case, we obtain $u(t)\in\mathscr{W}_{1}^{+}$ for any $t\in[0,\infty)$. By (2.3) and (4.1), we obtain

4.13 $$\begin{aligned} J(u_{0})>J \bigl(u(t) \bigr)={}&\frac{1}{q}I \bigl(u(t) \bigr)+ \biggl(\frac{1}{2}-\frac{1}{q} \biggr) \int_{\varOmega}\bigl\vert \Delta u(t) \bigr\vert ^{2} \,dx+\frac{1}{q^{2}} \int_{\varOmega}\bigl\vert u(t) \bigr\vert ^{q}\,dx \\ \geq{}& \biggl(\frac{1}{2}-\frac{1}{q} \biggr) \int_{\varOmega}\bigl\vert \Delta u(t) \bigr\vert ^{2} \,dx+\frac{1}{q^{2}} \int_{\varOmega}\bigl\vert u(t) \bigr\vert ^{q}\,dx. \end{aligned}$$

By $I(u(t))>0$, (2.4) and Lemma 2.1, there exists a $\overline{\lambda}_{3}>1$ such that $I(\overline{\lambda}_{3}u(t))=0$. We have

4.14 $$\begin{aligned} d\leq{}& J \bigl(\overline{\lambda}_{3}u(t) \bigr) \\ ={}&\frac{1}{q}I \bigl(\overline{\lambda}_{3}u(t) \bigr) + \biggl( \frac{1}{2}-\frac{1}{q} \biggr) \int_{\varOmega}\bigl\vert \Delta \bigl(\overline{ \lambda}_{3}u(t) \bigr) \bigr\vert ^{2}\,dx+ \frac{1}{q^{2}} \int _{\varOmega}\bigl\vert \overline{\lambda}_{3}u(t) \bigr\vert ^{q} \,dx \\ ={}& \biggl(\frac{1}{2}-\frac{1}{q} \biggr) \int_{\varOmega}\bigl\vert \Delta \bigl(\overline{ \lambda}_{3}u(t) \bigr) \bigr\vert ^{2}\,dx+ \frac{1}{q^{2}} \int _{\varOmega}\bigl\vert \overline{\lambda}_{3}u(t) \bigr\vert ^{q} \,dx \\ ={}& \biggl(\frac{1}{2}-\frac{1}{q} \biggr){\overline{ \lambda}_{3}}^{2} \int_{\varOmega}\bigl\vert \Delta u(t) \bigr\vert ^{2} \,dx+\frac{1}{q^{2}}{ \overline{\lambda }_{3}}^{q} \int_{\varOmega}\bigl\vert u(t) \bigr\vert ^{q}\,dx \\ ={}&{\overline{\lambda}_{3}}^{q} \biggl( \biggl( \frac{1}{2}-\frac{1}{q} \biggr) {\overline{\lambda}_{3}}^{2-q} \int_{\varOmega}\bigl\vert \Delta u(t) \bigr\vert ^{2} \,dx+\frac{1}{q^{2}} \int_{\varOmega}\bigl\vert u(t) \bigr\vert ^{q}\,dx \biggr) \\ \leq{}&{\overline{\lambda}_{3}}^{q} \biggl( \biggl( \frac{1}{2}-\frac{1}{q} \biggr) \int_{\varOmega}\bigl\vert \Delta u(t) \bigr\vert ^{2} \,dx+\frac{1}{q^{2}} \int_{\varOmega}\bigl\vert u(t) \bigr\vert ^{q}\,dx \biggr). \end{aligned}$$

Using (4.13) and (4.14), we have

$$\begin{aligned} d\leq{\overline{\lambda}_{3}}^{q}J(u_{0}), \end{aligned}$$

which implies that

4.15 $$\begin{aligned} \overline{\lambda}_{3}\geq \biggl(\frac{d}{J(u_{0})} \biggr)^{\frac{1}{q}}. \end{aligned}$$

It follows from (2.2) that

4.16 $$\begin{aligned} 0={}&I \bigl(\overline{\lambda}_{3}u(t) \bigr)= \int_{\varOmega}\bigl\vert \Delta \bigl(\overline{\lambda }_{3}u(t) \bigr) \bigr\vert ^{2}\,dx- \int_{\varOmega}\bigl\vert \overline{\lambda}_{3}u(t) \bigr\vert ^{q} \log \bigl\vert \overline{\lambda}_{3}u(t) \bigr\vert \,dx \\ ={}&{\overline{\lambda}_{3}}^{2} \int_{\varOmega}\bigl\vert \Delta u(t) \bigr\vert ^{2} \,dx-{\overline { \lambda}_{3}}^{q}\log\overline{ \lambda}_{3} \int_{\varOmega}\bigl\vert u(t) \bigr\vert ^{q}\,dx \\ &{}-{\overline{\lambda}_{3}}^{q} \int_{\varOmega}\bigl\vert u(t) \bigr\vert ^{q}\log \bigl\vert u(t) \bigr\vert \,dx \\ ={}&{\overline{\lambda}_{3}}^{q}I \bigl(u(t) \bigr)+{ \overline{ \lambda}_{3}}^{2} \int _{\varOmega}\bigl\vert \Delta u(t) \bigr\vert ^{2}\,dx-{\overline{ \lambda}_{3}}^{q} \int_{\varOmega}\bigl\vert \Delta u(t) \bigr\vert ^{2} \,dx \\ &{}-{\overline{\lambda}_{3}}^{q}\log\overline{ \lambda}_{3} \int_{\varOmega}\bigl\vert u(t) \bigr\vert ^{q}\,dx \\ ={}&{\overline{\lambda}_{3}}^{q}I \bigl(u(t) \bigr)+ \bigl({ \overline{\lambda }_{3}}^{2}-{\overline{ \lambda}_{3}}^{q} \bigr) \int_{\varOmega}\bigl\vert \Delta u(t) \bigr\vert ^{2} \,dx \\ &{}-{\overline{\lambda}_{3}}^{q}\log\overline{ \lambda}_{3} \int_{\varOmega}\bigl\vert u(t) \bigr\vert ^{q}\,dx. \end{aligned}$$

Using (4.15) and (4.16), we have

$$\begin{aligned} {\overline{\lambda}_{3}}^{q}I \bigl(u(t) \bigr)={}& \bigl({ \overline{\lambda}_{3}}^{q} -{\overline{ \lambda}_{3}}^{2} \bigr) \int_{\varOmega}\bigl\vert \Delta u(t) \bigr\vert ^{2} \,dx+{\overline { \lambda}_{3}}^{q}\log\overline{ \lambda}_{3} \int_{\varOmega}\bigl\vert u(t) \bigr\vert ^{q}\,dx \\ \geq{}& \bigl({\overline{\lambda}_{3}}^{q} -{\overline{ \lambda}_{3}}^{2} \bigr) \int_{\varOmega}\bigl\vert \Delta u(t) \bigr\vert ^{2} \,dx, \end{aligned}$$

which implies that

4.17 $$\begin{aligned} I \bigl(u(t) \bigr)\geq \bigl(1-{\overline{\lambda}_{3}}^{2-q} \bigr) \int_{\varOmega}\bigl\vert \Delta u(t) \bigr\vert ^{2} \,dx. \end{aligned}$$

It follows from (4.15) and (4.17) that

4.18 $$\begin{aligned} I \bigl(u(t) \bigr)\geq{}& \bigl(1-{\overline{ \lambda}_{3}}^{2-q} \bigr) \int_{\varOmega}\bigl\vert \Delta u(t) \bigr\vert ^{2} \,dx \\ \geq{}& \biggl[1- \biggl(\frac{d}{J(u_{0})} \biggr)^{{\frac{2}{q}}-1} \biggr] \int _{\varOmega}\bigl\vert \Delta u(t) \bigr\vert ^{2}\,dx \\ \geq{}&C_{23}^{-1} \biggl[1- \biggl(\frac{d}{J(u_{0})} \biggr)^{{\frac {2}{q}}-1} \biggr] \int_{\varOmega}\bigl\vert D u(t) \bigr\vert ^{2}\,dx \\ \geq{}&C_{23}^{-1}C_{24}^{-1} \biggl[1- \biggl(\frac{d}{J(u_{0})} \biggr)^{{\frac {2}{q}}-1} \biggr] \int_{\varOmega}\bigl\vert u(t) \bigr\vert ^{2}\,dx, \end{aligned}$$

where $C_{23}$ and $C_{24}$ are the Poincaré inequality constants. Hence, by (4.18), we obtain

4.19 $$\begin{aligned} I \bigl(u(t) \bigr)\geq{}&\frac{1}{3} \biggl[1- \biggl( \frac{d}{J(u_{0})} \biggr)^{{\frac {2}{q}}-1} \biggr] \int_{\varOmega}\bigl\vert \Delta u(t) \bigr\vert ^{2} \,dx \\ &{}+\frac{1}{3}C_{23}^{-1} \biggl[1- \biggl( \frac{d}{J(u_{0})} \biggr)^{{\frac {2}{q}}-1} \biggr] \int_{\varOmega}\bigl\vert D u(t) \bigr\vert ^{2}\,dx \\ &{}+\frac{1}{3}C_{23}^{-1}C_{24}^{-1} \biggl[1- \biggl(\frac{d}{J(u_{0})} \biggr)^{{\frac{2}{q}}-1} \biggr] \int_{\varOmega}\bigl\vert u(t) \bigr\vert ^{2}\,dx \\ \geq{}&C_{25} \biggl( \int_{\varOmega}\bigl\vert \Delta u(t) \bigr\vert ^{2} \,dx+ \int_{\varOmega}\bigl\vert D u(t) \bigr\vert ^{2}\,dx+ \int_{\varOmega}\bigl\vert u(t) \bigr\vert ^{2}\,dx \biggr) \\ ={}&C_{25} \bigl\Vert u(t) \bigr\Vert _{H_{0}^{2}(\varOmega)}^{2}, \end{aligned}$$

where

$$\begin{aligned} C_{25}={}&\max \biggl\{ \frac{1}{3} \biggl[1- \biggl( \frac{d}{J(u_{0})} \biggr)^{{\frac{2}{q}}-1} \biggr], \frac{C_{21}^{-1}}{3} \biggl[1- \biggl(\frac {d}{J(u_{0})} \biggr)^{{\frac{2}{q}}-1} \biggr], \\ &\frac{C_{21}^{-1}C_{22}^{-1}}{3} \biggl[1- \biggl(\frac{d}{J(u_{0})} \biggr)^{{\frac{2}{q}}-1} \biggr] \biggr\} . \end{aligned}$$

Integrating the $I(u(s))$ with respect to *s* over $(t,T)$ and using the embedding $H_{0}^{2}(\varOmega)\hookrightarrow L^{2}(\varOmega)$, we obtain

4.20 $$\begin{aligned} \int_{t}^{T}I \bigl(u(s) \bigr)\,ds={}&{-} \int_{t}^{T} \int_{\varOmega}u_{s}(s)u(s)\,dx\,ds =-\frac{1}{2} \int_{\varOmega}u(T)^{2}\,dx+\frac{1}{2} \int_{\varOmega}u(t)^{2}\,dx \\ \leq{}&\frac{1}{2} \int_{\varOmega}u(t)^{2}\,dx \\ \leq{}&\frac{1}{2}C_{26}^{2} \bigl\Vert u(t) \bigr\Vert _{H_{0}^{2}(\varOmega)}^{2}, \end{aligned}$$

where $C_{26}$ is the embedding constant. From (4.19) and (4.20), we have

4.21 $$\begin{aligned} \int_{t}^{T}C_{25} \bigl\Vert u(t) \bigr\Vert _{H_{0}^{2}(\varOmega)}^{2}\,ds \leq\frac{1}{2}C_{26}^{2} \bigl\Vert u(t) \bigr\Vert _{H_{0}^{2}(\varOmega)}^{2},\quad \mbox{for all }t\in[0,T]. \end{aligned}$$

Let $T\rightarrow\infty$ in (4.21), we can get

4.22 $$\begin{aligned} \int_{t}^{\infty} \bigl\Vert u(t) \bigr\Vert _{H_{0}^{2}(\varOmega)}^{2} \,ds \leq\frac{1}{2}C_{25}^{-1}C_{26}^{2} \bigl\Vert u(t) \bigr\Vert _{H_{0}^{2}(\varOmega)}^{2}. \end{aligned}$$

From Lemma 2.4, we have

$$\begin{aligned} \bigl\Vert u(t) \bigr\Vert _{H_{0}^{2}(\varOmega)}^{2}\leq \bigl\Vert u(0) \bigr\Vert _{H_{0}^{2}(\varOmega)}^{2}e^{1-\frac{2t}{C_{25}^{-1}C_{26}^{2}}},\quad t\in[0, \infty). \end{aligned}$$

The above inequality implies that the solution $u(t)$ decays exponentially. □

## Blow up

### Theorem 5.1

*If* $u_{0}\in\mathscr{W}_{1}^{-}$, *the unique local weak solution* $u(t)$ *of the problem* (1.1) *blows up in finite time*, *i*.*e*., *there exists a* $T_{*}>0$ *such that*

$$\begin{aligned} \lim_{t\to T_{*}^{-}} \int_{\varOmega}\bigl\vert u(t) \bigr\vert ^{2}\,dx= \infty. \end{aligned}$$

### Proof

Since $u_{0}\in\mathscr{W}_{1}^{-}$, it follows from the local existence that there exists a unique local weak solution $u(t)$ of the problem (1.1) such that

5.1 $$\begin{aligned} \int_{0}^{t} \int_{\varOmega}u_{s}^{2}\,dx\,ds+J \bigl(u(t) \bigr)\leq J(u_{0})< d,\quad t\in[0,T_{\mathrm{max}}]. \end{aligned}$$

Next, we prove $u(t)\in\mathscr{W}_{1}^{-}$ for $t\in[0,T_{\mathrm{max}}]$. We assume $u(t)$ leaves $\mathscr{W}_{1}^{+}$ at time $t=t_{1}$, then there exists a sequence $\{t_{n}\}$ such that $I(u(t_{n}))\leq0 $ as $t_{n}\rightarrow t_{1}^{-}$. It follows from lower semicontinuity of $L^{2}$ norm that

5.2 $$\begin{aligned} I \bigl(u(t_{1}) \bigr)\leq\liminf_{n \to\infty}I \bigl(u(t_{n}) \bigr)\leq0. \end{aligned}$$

We have $I(u(t_{1}))=0$ according to $u(t_{1})\notin\mathscr{W}_{1}^{+}$. By (2.4) and (5.1), we have

$$\begin{aligned} d=\inf_{u\in\mathscr{N}}J(u)\leq J \bigl(u(t_{1}) \bigr)< d, \end{aligned}$$

which is a contradiction. So, $u(t)\in\mathscr{W}_{1}^{-}$ for $t\in [0,T_{\mathrm{max}}]$. Next, we will study that $u(t)$ blows up in finite time by contradiction. Thus, we assume $u(t)$ is global. We contract a function $\varPhi:[0,\infty)\rightarrow\mathbb {R}^{+}$, and

5.3 $$\begin{aligned} \varPhi(t)= \int_{0}^{t} \int_{\varOmega}u^{2}\,dx\,ds. \end{aligned}$$

We can easily obtain

5.4 $$\begin{aligned} \varPhi'(t)= \int_{\varOmega}u^{2}\,dx. \end{aligned}$$

By (2.2) and (5.4), we have

5.5 $$\begin{aligned} \varPhi''(t)=2 \int_{\varOmega}uu_{t}\,dx=2 \int_{\varOmega} \vert u \vert ^{q-2}u\log \vert u \vert \,dx-2 \int_{\varOmega} \vert \Delta u \vert ^{2}\,dx =-2I(u). \end{aligned}$$

From $u(t)\in\mathscr{W}_{1}^{-}$ and (5.5), we can obtain

5.6 $$\begin{aligned} \varPhi''(t)>0. \end{aligned}$$

Thus, it follows from $u_{0}\in\mathscr{W}_{1}^{-}$ and (5.4) that

5.7 $$\begin{aligned} \varPhi'(t)\geq\varPhi'(0)= \int_{\varOmega}u_{0}^{2}\,dx>0. \end{aligned}$$

Using the Hölder inequality and combining (5.5), we have

5.8 $$\begin{aligned} \frac{1}{4} \bigl(\varPhi'(t)- \varPhi'(0) \bigr)^{2}&= \frac{1}{4} \biggl( \int_{0}^{t}\varPhi''(s) \,ds \biggr)^{2} = \biggl( \int_{0}^{t} \int_{\varOmega}uu_{s}\,dx\,ds \biggr)^{2} \\ &\leq \int_{0}^{t} \int_{\varOmega}u^{2}\,dx\,ds \int_{0}^{t} \int_{\varOmega}u_{s}^{2}\,dx\,ds. \end{aligned}$$

By (2.3) and (5.5), we have

5.9 $$\begin{aligned} \varPhi''(t)={}&{-}2I(u) =-2qJ(u) +2q \biggl(\frac{1}{2}-\frac{1}{q} \biggr) \int_{\varOmega} \vert \Delta u \vert ^{2}\,dx+ \frac{2}{q} \int_{\varOmega} \vert u \vert ^{q}\,dx \\ ={}&{-}2qJ(u_{0})+2q \int_{0}^{t} \int_{\varOmega}u_{s}^{2}\,dx\,ds \\ &{} +2q \biggl(\frac{1}{2}-\frac{1}{q} \biggr) \int_{\varOmega} \vert \Delta u \vert ^{2}\,dx + \frac{2}{q} \int_{\varOmega} \vert u \vert ^{q}\,dx. \end{aligned}$$

Since $u(t)\in\mathscr{W}_{1}^{-}$, $I(u(t))<0$. By Lemma 2.1, there exists a $\overline{\lambda}_{4}$, $0<\overline{\lambda}_{4}<1$ such that $I(\overline{\lambda}_{4}u(t)))=0$. It follows from (2.3) and (2.4) that

5.10 $$\begin{aligned} d={}&\inf_{u\in\mathscr{N}}J(u)\leq J \bigl(\overline{ \lambda}_{4}u(t) \bigr) \\ ={}&\frac{1}{q}I \bigl(\overline{\lambda}_{4}u(t) \bigr) + \biggl( \frac{1}{2}-\frac{1}{q} \biggr) \int_{\varOmega}\bigl\vert \Delta \bigl(\overline{ \lambda}_{4}u(t) \bigr) \bigr\vert ^{2}\,dx + \frac{1}{q^{2}} \int_{\varOmega}\bigl\vert \overline{\lambda}_{4}u(t) \bigr\vert ^{q} \,dx \\ ={}& \biggl(\frac{1}{2}-\frac{1}{q} \biggr) \int_{\varOmega}\bigl\vert \Delta \bigl(\overline{ \lambda}_{4}u(t) \bigr) \bigr\vert ^{2}\,dx + \frac{1}{q^{2}} \int_{\varOmega}\bigl\vert \overline{\lambda}_{4}u(t) \bigr\vert ^{q} \,dx \\ ={}&\overline{\lambda}_{4}^{2} \biggl(\frac{1}{2}- \frac{1}{q} \biggr) \int_{\varOmega}\bigl\vert \Delta u(t) \bigr\vert ^{2} \,dx +\overline{ \lambda}_{4}^{q}\frac{1}{q^{2}} \int_{\varOmega}\bigl\vert u(t) \bigr\vert ^{q}\,dx \\ \leq{}& \biggl(\frac{1}{2}-\frac{1}{q} \biggr) \int_{\varOmega}\bigl\vert \Delta u(t) \bigr\vert ^{2} \,dx +\frac{1}{q^{2}} \int_{\varOmega}\bigl\vert u(t) \bigr\vert ^{q}\,dx. \end{aligned}$$

Combining (5.9) with (5.10), we have

5.11 $$\begin{aligned} \varPhi''(t) ={}&{-}2qJ(u_{0})+2q \int_{0}^{t} \int_{\varOmega}u_{s}^{2}\,dx\,ds +2q \biggl( \frac{1}{2}-\frac{1}{q} \biggr) \int_{\varOmega} \vert \Delta u \vert ^{2}\,dx + \frac{2}{q} \int_{\varOmega} \vert u \vert ^{q}\,dx \\ ={}&{-}2qJ(u_{0})+2q \int_{0}^{t} \int_{\varOmega}u_{s}^{2}\,dx\,ds \\ &{}+2q \biggl[ \biggl(\frac{1}{2}-\frac{1}{q} \biggr) \int_{\varOmega}\bigl\vert \Delta u(t) \bigr\vert ^{2} \,dx +\frac{1}{q^{2}} \int_{\varOmega}\bigl\vert u(t) \bigr\vert ^{q}\,dx \biggr] \\ \geq{}&2q \bigl(d-J(u_{0}) \bigr)+2q \int_{0}^{t} \int_{\varOmega}u_{s}^{2}\,dx\,ds. \end{aligned}$$

Using (5.3), (5.8) and (5.11), we have

5.12 $$\begin{aligned} \varPhi(t)\varPhi''(t)={}& \int_{0}^{t} \int_{\varOmega}u^{2}\,dx\,ds\varPhi''(t) \\ \geq{}& \int_{0}^{t} \int_{\varOmega}u^{2}\,dx\,ds \biggl[2q \bigl(d-J(u_{0}) \bigr)+2q \int_{0}^{t} \int_{\varOmega}u_{s}^{2}\,dx\,ds \biggr] \\ \geq{}&\varPhi(t)2q \bigl(d-J(u_{0}) \bigr)+\frac{q}{2} \bigl( \varPhi'(t)-\varPhi'(0) \bigr)^{2}. \end{aligned}$$

We fix a $t_{2}>0$. It follows from (5.7) that we have

5.13 $$\begin{aligned} \varPhi(t)\geq\varPhi(t_{2})= \int_{0}^{t_{2}} \int_{\varOmega}u^{2}\,dx\,ds\geq t_{2} \int_{\varOmega}u_{0}^{2}\,dx>0,\quad \mbox{for }t \in[t_{2},\infty). \end{aligned}$$

Hence, by (5.12) and (5.13), we have

5.14 $$\begin{aligned} \varPhi(t)\varPhi''(t)- \frac{q}{2} \bigl(\varPhi'(t)-\varPhi'(0) \bigr)^{2} \geq{}&\varPhi(t)2q \bigl(d-J(u_{0}) \bigr) \\ \geq{}& t_{2} \int_{\varOmega}u_{0}^{2}\,dx >0, \quad \mbox{for }t \in[t_{2},\infty). \end{aligned}$$

We choose $T>t_{2}$ sufficiently large and contract a function $\varPsi (t)$ as follows:

5.15 $$\begin{aligned} \varPsi(t)=\varPhi(t)+(T-t) \int_{\varOmega}u_{0}^{2}\,dx,\quad t \in[t_{2},T]. \end{aligned}$$

From (5.13) and (5.15), we can easily see that for any $t\in [t_{2},T]$, $\varPsi(t)\geq\varPhi(t)>0$ holds. It follows from (5.4) and (5.14) that, for any $t\in [t_{2},T]$, $\varPsi'(t)=\varPhi'(t)-\varPhi'(0)$ holds, thus we also have $\varPsi''(t)=\varPhi''(t)>0$ from (5.6). Thus, we can obtain from (5.14)

5.16 $$\begin{aligned} \varPsi(t)\varPsi''(t)- \frac{q}{2}\varPsi'(t)^{2} \geq{}&\varPhi(t) \varPhi''(t)+\frac{q}{2} \bigl( \varPhi'(t)-\varPhi'(0) \bigr)^{2} \\ \geq{}&\varPhi(t)2q \bigl(d-J(u_{0}) \bigr) \\ \geq{}& t_{2} \int_{\varOmega}u_{0}^{2}\,dx>0, \end{aligned}$$

for $t\in[t_{2},T]$. Let $\chi(t)=\varPsi(t)^{-\frac{q-2}{2}}$. Thus,

5.17 $$\begin{aligned} \chi'(t)={-\frac{q-2}{2}} \varPsi(t)^{-\frac{q}{2}} \varPsi'(t). \end{aligned}$$

From (5.16) and (5.17), we have

5.18 $$\begin{aligned} \chi''(t)={}&{\frac{q(q-2)}{4}} \varPsi(t)^{-\frac{q+2}{2}}\varPsi'(t)^{2} {- \frac{q-2}{2}}\varPsi(t)^{-\frac{q}{2}}\varPsi''(t) \\ ={}&\frac{q-2}{2}\varPsi(t)^{-\frac{q+2}{2}} \biggl[\frac{q}{2}\varPsi '(t)^{2}-\varPsi(t)\varPsi''(t) \biggr] < 0, \end{aligned}$$

for $t\in[t_{2},T]$. This shows that, for any sufficiently large $T>t_{2}$, $\chi(t)$ is a concave function in $[t_{2},T]$. $\chi(t_{2})>0$ and $\chi ''(t_{2})<0$, so there exists a finite time $T_{*}>t_{2}>0$ such that

$$\begin{aligned} \lim_{t \to T_{*}^{-}}\chi(t)=0, \end{aligned}$$

which implies

$$\begin{aligned} \lim_{t \to T_{*}^{-}}\varPsi(t)=\infty. \end{aligned}$$

Hence, we have

$$\begin{aligned} \lim_{t \to T_{*}^{-}} \int_{0}^{t} \int_{\varOmega}u^{2}\,dx\,ds=\infty, \end{aligned}$$

i.e.,

$$\begin{aligned} \lim_{t \to T_{*}^{-}} \int_{\varOmega}u^{2}\,dx=\infty. \end{aligned}$$

This is a contradiction to our assumption. □
